# Centralization of cleft care in the UK. Part 6: a tale of two studies

**DOI:** 10.1111/ocr.12111

**Published:** 2015-11-16

**Authors:** A R Ness, A K Wills, A Waylen, R Al-Ghatam, T E M Jones, R Preston, A J Ireland, M Persson, J Smallridge, A J Hall, D Sell, J R Sandy

**Affiliations:** National Institute for Health Research (NIHR) Biomedical Research Unit in Nutrition, Diet and Lifestyle at the University Hospitals Bristol NHS Foundation Trust and the University of BristolBristol, UK; School of Oral and Dental Sciences, University of BristolBristol, UK; Musgrove Park HospitalTaunton, UK; Cleft Lip and Palate AssociationLondon, UK; Centre for Appearance Research, University of the West of EnglandBristol, UK; South Thames’ Cleft Unit, Guy’s and St Thomas HospitalLondon, UK; Cleft Net East Cleft Network, Addenbrooke’s HospitalCambridge, UK; Children’s Hearing Centre, University Hospitals Bristol NHS Foundation TrustBristol, UK; Centre for Child and Adolescent Health, School of Social and Community Medicine, University of BristolBristol, UK; Speech and Language Therapy Department and Centre for Outcomes and Experience Research in Children’s Health, Illness and Disability (ORCHID), Great Ormond Street Hospital NHS Foundation TrustLondon, UK

**Keywords:** Cleft Lip, Cleft Palate, Cross-Sectional Studies

## Abstract

**Objectives:**

We summarize and critique the methodology and outcomes from a substantial study which has investigated the impact of reconfigured cleft care in the United Kingdom (UK) 15 years after the UK government started to implement the centralization of cleft care in response to an earlier survey in 1998, the Clinical Standards Advisory Group (CSAG).

**Setting and Sample Population:**

A UK multicentre cross-sectional study of 5-year-olds born with non-syndromic unilateral cleft lip and palate. Data were collected from children born in the UK with a unilateral cleft lip and palate between 1 April 2005 and 31 March 2007.

**Materials and Methods:**

We discuss and contextualize the outcomes from speech recordings, hearing, photographs, models, oral health and psychosocial factors in the current study. We refer to the earlier survey and other relevant studies.

**Results:**

We present arguments for centralization of cleft care in healthcare systems, and we evidence this with improvements seen over a period of 15 years in the UK. We also make recommendations on how future audit and research may configure.

**Conclusions:**

Outcomes for children with a unilateral cleft lip and palate have improved after the introduction of a centralized multidisciplinary service, and other countries may benefit from this model. Predictors of early outcomes are still needed, and repeated cross-sectional studies, larger longitudinal studies and adequately powered trials are required to create a research-led evidence-based (centralized) service.

## Introduction

In this supplement, we report on the results of a UK-wide cross-sectional survey of 5-year-old children with unilateral cleft lip and palate conducted between January 2011 and December 2012 – Cleft Care UK (CCUK) 1–5. We attempted as far as possible to follow the design of a previous survey conducted fifteen years ago that recruited from a similar target population of children with unilateral cleft lip and palate– the Clinical Standards Advisory Group (CSAG) study 6–11. Much has changed since this first survey was conducted. The 57 centres providing care for children with cleft lip and palate have reduced to 11 centres or managed clinical networks. Care in these centres is provided by multidisciplinary teams, and the surgeons in these teams operate on at least 35 cases each year 12–14. Preliminary studies suggest that outcomes have improved but these reports either were regional (rather than national), were specific to a single outcome or had incomplete data 15–17. We report here the results of a comprehensive nationwide survey and directly compare these results with those prior to centralization.

## A tale of two studies

We were not able to replicate the previous survey exactly. We used the same inclusion criteria and measured the same attributes in the study children. But the children we studied fifteen years later were younger (despite using the same target age range), and some of the approaches to measurement have changed. Orthodontists now sometimes use photographs in place of study models, speech and language therapists assess speech using a modified protocol, and psychologists have changed the questions they ask. The similarities and differences between the two studies are summarized in Table1 and described in detail in the first paper in this supplement 1. Despite these differences, we believe that these studies are similar enough to allow us to describe changes in care and outcome over this time frame and thus to evaluate the impact of the move to a centralized multidisciplinary service.

**Table 1:** Comparison of methods and demographics between the Cleft Care UK (CCUK) study and the Clinical Standards Advisory Group (CSAG) study

	CCUK 2012	CSAG 1998
Methods		
Type of activity	Research	Audit
People collecting key outcomes	Mainly local teams	Study team
Measures		
Appearance	Better quality digital images	Photographs
Dento-alveolar arch relationships	Study models and photographs	Study models
Oral health	British Association for the Study of Community Dentistry (BASCD) calibrated dental examination by consultant paediatric dentist	BASCD calibrated dental examination by an orthodontist
Hearing	Pure tone audiometry, tympanometry and otology assessment	Pure tone audiometry and otology assessment
Speech assessment	Cleft Audit Protocol for Speech – Augmented (CAPS-A)	Cleft Audit Protocol for Speech (CAPS)
Psychology assessment	Modified psychosocial questionnaire, 8 items; self-confidence response was 0–10 (0 = very negative effect; 5 = no difference; 10 = very positive effect)	Psychosocial questionnaire, 18 items; self-confidence response was yes/no
Demographics		
Year of birth	2005–2007	1989–1991
Eligible	359	326
Number recruited and response rate	268 (75%)	239 (73%)
Age (median and interquartile range)	5.5 (5.4–5.7)	6.4 (5.9–6.9)
Number of boys (percentage)	181 (67.5%)	159 (66.5%)

## Summary of findings

The treatment offered to children has changed over the last 15 years. The range of surgical procedures used is less varied, hearing aids are used more often, and grommets placed less frequently. Overall outcomes have improved. There have been marked improvements in dento-alveolar arch relationships and in speech, whereas the prevalence of dental caries and hearing loss is unchanged. These differences are summarized in Table2 and described in more detail in the results papers in this supplement 2–5. Though much improved, comparative data from other centres on dento-alveolar arch relationships and speech outcomes suggest these are still not as good as those achieved in the best centres in Europe 18–20. Further, there are still a proportion of children who do badly with up to 20% having poor results for important outcomes such as dento-alveolar arch relationships and intelligibility/distinctiveness of speech. The numbers with poor outcomes are summarized in Table3 and described in more detail in the results papers in this supplement 2–5.

**Table 2:** Comparison of good outcomes between the Cleft Care UK (CCUK) study and the Clinical Standards Advisory Group (CSAG) study

	CCUK	CSAG	*P*-value
Structural outcomes			
Facial appearance (% good or excellent)	36	32	0.107*
Dento-alveolar relationships (% good or excellent)	53†	30	<0.001‡
Oral health			
Caries-free (dmft = 0) (%)	48	45	0.6‡
Hearing			
None or mild hearing loss in better ear (%)	78	79	0.7‡
Speech			
No hypernasality (%)	90	82	0.018‡
Intelligibility/distinctiveness (% normal)	56	20	<0.001‡
Psychosocial outcome			
Child’s self-confidence not affected (%)	92	81	<0.001‡

* Mixed effects logistic regression to account for the non-independence of observations from multiple observers.

† This is based on the 198 children with models as the 49 children with photographs were excluded.

‡ Chi-squared test.

**Table 3:** Comparison of poor outcomes between the Cleft Care UK (CCUK) study and the Clinical Standards Advisory Group (CSAG) study

	CCUK	CSAG	*P*-value
Structural outcomes			
Facial appearance (% poor or very poor)	22	28	0.013*
Dento-alveolar relationships (% poor or very poor)	19†	36	<0.001‡
Oral health			
Caries present (dmft ≥1) (%)	52	55	0.6‡
Hearing			
Moderate or worse hearing loss in better ear (%)	22	21	0.7‡
Speech			
Hypernasality (%)	10	18	0.018‡
Intelligibility/distinctiveness (% just intelligible or less)	17	19	0.6‡
Psychosocial outcome			
Child’s self-confidence affected (%)	8	19	<0.001‡

* Mixed effects logistic regression to account for the non-independence of observations from multiple observers.

† This is based on the 198 children with models as the 49 children with photographs were excluded.

‡ Chi-squared test.

## Other benefits of this programme of cleft research

This second cross-sectional survey was part of a larger programme of work funded by the UK National Institute for Health Research (NIHR). We ran a series of research workshops to design the study that also triggered a James Lind Alliance initiative 21 and a health talk project 22 in children with cleft lip and palate. We completed several systematic reviews on treatment for children with cleft lip and palate 23–25. We conducted a survey of cleft centres that has described the service provision 12,13 and the process of centralization 26. We have been able to offer training opportunities. Three students have already completed taught doctorates using data collected as part of this cross-sectional survey and the survey of the centres. We have strengthened patient and public involvement among people with cleft by disseminating our work through the Cleft Lip and Palate Association and by running a workshop focussing on patient involvement. Our study (and programme) has thus paved the way for future randomized trials and observation studies by reviewing the evidence and building research capacity in cleft centres in the UK.

## Implications for practice

Our data show that a centralized multidisciplinary service improves outcomes albeit that some areas of cleft care still require improvement. This study will provide evidence for cleft teams to argue with commissioners for increased resource. In our view, centralized multidisciplinary services should be introduced in all countries. Local comprehensive surveys are not required to justify or guide this change. Earlier UK outcomes described in the original CSAG study were poor, and it was argued that this was because there was no centralized service. This does not prove at all that small centres with a low case load but very well-organized care are likely to have poor outcomes. The issue is that these small centres will have great difficulty in proving the quality of their outcomes because of a lack of statistical power. These arguments have been well rehearsed elsewhere with strong evidence to counter the continuation of low volume operating 10. However, it is not clear what the key component (or components) of centralization is. Is it the improvements in surgical training? Is it the increase in number of operations? Is it the implementation of multidisciplinary team working? Is it the creation of an audit culture that encourages reflective practice? Further analyses of the data in these two cross-sectional studies and future studies may refine our understanding, but this should not delay plans to rationalize services. It is also unclear how we should monitor outcome post-centralization. It would be useful to have process measures that predict outcome at age five, or earlier outcome measures that can be used before the age of 5 years or both. In the UK, we rely on data collected at audit clinics when the child is 5 years old. These data therefore reflect outcome sometime after the primary surgery, and the numbers treated in any one centre are small. Obtaining standardized measures from all centres routinely is challenging and expensive 17. In our view, the best approach is to encourage audit clinics locally (where these do not already happen) that provide training and encourage review of personal practice – this will certainly be able to detect extreme variations in outcome – and to carry out intermittent comprehensive surveys nationally that describe care and outcomes. It is essential to continue to participate in international comparative studies and to be mindful of the better European centres where care is still being delivered at higher standards with better outcomes.

## Implications for research

We plan to conduct further analyses of these data to look at the impact of centre characteristics such as size, time to centralization, surgical throughput and multidisciplinary working on outcome. We also intend to look at predictors of specific outcomes in an attempt to quantify the role of individual and treatment factors that predict both good and poor outcomes. We will also describe the costs incurred by families and people’s choice preferences. This cross-sectional study is a resource, and we are trying to encourage future collaborations to ensure it is fully exploited. We are currently creating a detailed data dictionary and formalizing access arrangements. A further national cross-sectional survey should be considered in 5–10 years to confirm that there have been further improvements in service provision and outcome such that cleft care in the UK is the best in Europe. Longitudinal studies to describe trajectories of children with cleft and to identify early outcome measures or predictors of outcome would be valuable. Well-designed adequately powered trials informed by the priorities identified through the recent James Lind Alliance initiative should be conducted 21. There is also now an opportunity for the cleft teams in the UK to participate in larger international studies of outcomes and to participate in clinical trials. These collaborations are key with a low incidence anomaly as all aspects of aetiology and care will benefit from increasing sample sizes. The diversity of genetic and environmental factors in the causation of clefting will only be explored through multicentre collaborations and international epidemiological approaches.

## The challenges of clinical research in cleft

The evidence base to inform treatment of children with cleft lip and palate is limited. There are few well-designed adequately powered randomized trials or prospective observational studies. Clinical research should be easier in a centralized service particularly in a state-funded health system on a small heavily populated island like the UK. We were able to recruit children to this study within a narrower age range than previously, but our response rates were similar. Furthermore, we faced challenges trying to collect data that were directly comparable with the previous study. Families do have further to travel to reach a cleft centre, and this may have reduced their willingness to come to clinic and to complete questionnaires. Interestingly, families did not report that it is any more difficult to attend the cleft centre 5. We decided to run this project as research rather than audit. This allowed us to obtain consent to carry out follow-up through record linkage, to collect additional data, to standardize and control data quality and to be able to contact families about participation in other studies. We had to navigate research approval processes which led to delays 27, we incurred extra costs, and we had to obtain consent from the parents and assent from the children. Few families declined to participate in the research project, but response rates for self-completed questionnaires were disappointing. So, our experience suggests that a centralized service does make it easier to conduct multicentre clinical research but that there is room for improvement. Future research projects need to consider strategies to reach and study socially disadvantaged groups who are less likely to come to clinics and complete questionnaires 28. The challenge is to build research understanding, expertise and capacity in teams. If this challenge is met, there is an opportunity to strengthen the evidence base to inform treatment decisions for children with cleft lip and palate 29.

## Conclusions

Outcomes for children with a unilateral cleft lip and palate have improved after the introduction of a centralized multidisciplinary service. This process of centralization should be introduced in other countries with a less centralized service for children with clefts. Further analyses will be conducted to explore centre-level effects and individual and treatment factors that influence outcome. There is a need for earlier outcome measures or predictors of outcome that could be used to audit practice and monitor service quality in a more timely fashion. Repeated cross-sectional studies, larger longitudinal studies and adequately powered trials are required to create the research-led evidence-based (centralized) service that children born with cleft lip and palate deserve.
